# New Polyvinyl Alcohol/Succinoglycan-Based Hydrogels for pH-Responsive Drug Delivery

**DOI:** 10.3390/polym15143009

**Published:** 2023-07-11

**Authors:** Jae-pil Jeong, Kyungho Kim, Jaeyul Kim, Yohan Kim, Seunho Jung

**Affiliations:** Department of Bioscience and Biotechnology, Konkuk University, Seoul 05029, Republic of Korea

**Keywords:** succinoglycan, polyvinyl alcohol, freeze–thaw, hydrogel, pH-responsive drug delivery

## Abstract

We fabricated new hydrogels using polyvinyl alcohol (PVA) and succinoglycan (SG) directly isolated and obtained from *Sinorhizobium meliloti* Rm 1021 via the freeze–thaw method. Both the composition of the hydrogels and the freeze–thaw cycles were optimized to maximize the swelling ratio for the preparation of the PVA/SG hydrogels. During the optimization process, the morphology and conformational change in the hydrogel were analyzed by scanning electron microscopy, rheological measurements, and compressive tests. An optimized hydrogel with a maximum swelling ratio of 17.28 g/g was obtained when the composition of PVA to SG was 50:50 (PVA/SG 50/50) and the total number of freeze–thaw cycles was five. The PVA/SG 50/50 hydrogel had the largest pore with 51.24% porosity and the highest cross-over point (28.17%) between the storage modulus (G′) and the loss modulus (G″). The PVA/SG 50/50 hydrogel showed improved thermal stability owing to its interaction with thermally stable SG chains. The improvement in the thermal stability was confirmed by thermogravimetric analysis and differential scanning calorimetry. In addition, the PVA/SG 50/50 hydrogel showed differential drug release according to the corresponding pH under acidic conditions of pH 1.2 and slightly basic conditions of pH 7.4. Furthermore, the cell viability test on the HEK-293 cell line for that hydrogel demonstrated that the PVA/SG 50/50 hydrogel was non-toxic and biocompatible. Therefore, this hydrogel could be a potential scaffold capable of pH-responsive drug delivery for chronic wound dressing applications.

## 1. Introduction

In the biomedical field, research related to the efficient delivery of drugs for various diseases has been conducted for several decades. For that purpose, various drug delivery systems using liposomes, nanoparticles, and hydrogels have been studied [1]. Among them, hydrogels have been widely used due to their good flexibility, softness, elasticity, water absorption ability, non-toxicity, biodegradability, and biocompatibility. They fabricate a 3D network through interactions connected with physical crosslinking or chemical crosslinking. Natural polymers like polysaccharides and synthetic polymers are usually used for this purpose [1]. The advantage of a hydrogel as a drug delivery system is that it is possible to control drug encapsulation and the delivery rate. Furthermore, another advantage of hydrogels is that they can control the drug delivery rate and encapsulation according to changes in the external environment, such as temperature or pH, depending on the characteristics of the polymer [2]. Based on those benefits, hydrogels have been used in the biomedical field, including drug delivery systems and tissue engineering.

Polyvinyl alcohol (PVA) is a synthetic polymer with a water-soluble, semi-crystalline structure with many hydroxyl groups that can easily form intermolecular hydrogen bonds [3,4]. PVA has been considered as one of the key materials used for hydrogels because of its excellent biocompatibility and mechanical properties. PVA-based hydrogels can be fabricated through chemical and physical crosslinking. The chemical crosslinking of PVA hydrogels can be constructed with the help of crosslinking agents such as glutaraldehyde [5], glyoxal [6], and borate [7]. Physical crosslinking of PVA can occur with repeated freeze–thaw cycles, inducing crystallization and constructing interconnected porous structures between PVA networks [8]. A hydrogel obtained by the freeze–thaw method has the advantage of minimizing the toxicity of the hydrogel itself by removing the incorporation of a chemical crosslinking agent [9].

In preparing a PVA-based hydrogel, PVA polymers are usually combined with other polymers with high-water uptake capabilities such as polysaccharides, proteins, and other synthetic polymers due to the lack of swelling ability of PVA hydrogels [10]. Polysaccharides, one of the abundant natural polymers, have been widely used as a material for hydrogels because of their own biocompatibility, non-immunogenicity, and functional versatility [11]. Polysaccharides can be derived from animals, plants, algae, and microorganisms. Compared with other sources, microbial polysaccharides have advantages with respect to being economical, easy to handle, and having short production cycles [12]. In addition to these merits, microbial polysaccharides have been applied in the food and biomedical fields because of their physical properties and pharmaceutical-related actions [13,14,15].

Succinoglycan (SG) is an extracellular polysaccharide isolated directly from soil microorganisms such as *Sinorhizobium* and *Agrobacterium* species [16,17]. Its composition consists of octasaccharide repeating units of seven glucoses and one galactose residue. SG has chemical substituents such as pyruvyl, succinyl, and acetyl groups [16] and has been widely used in various industries such as food and cosmetics based on its high viscosity and thermal stability [18]. Recently, the application of SG has expanded to the biomedical field according to its intrinsic biological and antibacterial effects [19,20,21]. Some reports on SG as a potential scaffold for drug delivery carriers have been published [22,23]. However, no studies have been reported on drug delivery systems using PVA/SG hydrogels based on the thermal stability and pH responsiveness of SG and the good mechanical properties of PVA.

In this study, we fabricated a hydrogel using PVA and SG via the freeze–thaw method. The effective factors such as the composition of the hydrogel and the freeze–thaw cycle were optimized to maximize the swelling capacity. After the optimization process, the characterization of the selected hydrogel was investigated to assess the change in conformation and thermal stability. The release profile of 5-fluorouracil (5-FU) from the hydrogel was monitored to investigate the pH-responsive drug release pattern, and the cytotoxicity was evaluated with the WST-8 assay.

## 2. Materials and Methods

### 2.1. Materials

The bacterial strain (*Sinorhizobium meliloti* Rm1021) was supplied by the Microbial Carbohydrate Resource Bank (MCRB) at Konkuk University (Seoul, Republic of Korea). Polyvinyl alcohol (PVA) (Mw ~ 89,000–98,000) and 5-fluorouracil (5-FU) were purchased from Sigma-Aldrich (Steinheim, Germany). Dulbecco’s minimum Eagle’s medium (DMEM), fetal bovine serum (FBS), trypsin/ethylenediaminetetraacetic acid (EDTA) solution, antibiotic/antimycotic solution, phosphate-buffered saline (PBS), and other materials used in the cell culture experiment were purchased from GIBCO™ (Gaithersburg, MD, USA). All other chemicals were of analytical grade and used without further purification.

### 2.2. Isolation of SG

The preparation of SG was performed as previously described [24]. *Sinorhizobium meliloti* Rm1021 was cultured at 30 °C for 7 days in a GMS (glutamic acid–mannitol–salt) medium. The pH of all media was adjusted to 7.00 using 1M NaOH solution. After 7 days, the cultured medium was centrifuged to obtain the supernatant, and the obtained supernatant was evaporated to reduce its volume. The SG was precipitated by adding 3 volumes of ethanol to the supernatant. The precipitated SG was dialyzed in distilled water for 5 days (MWCO: 12–14 kDa).

### 2.3. Synthesis of the PVA/SG Hydrogel

**Scheme 1:**
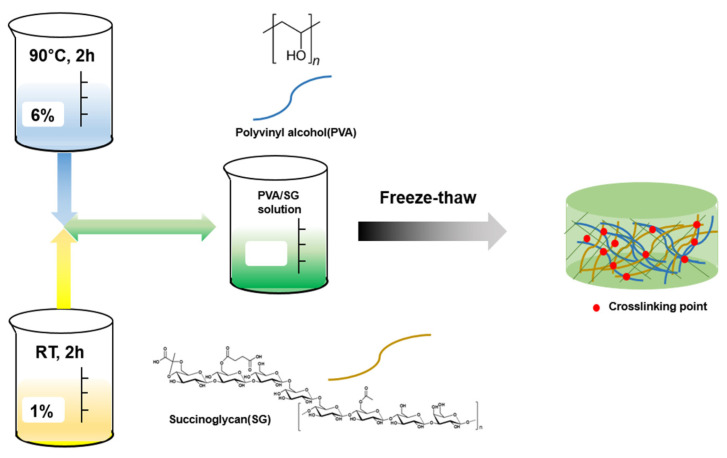
Illustration of the procedure for preparing the PVA/SG hydrogel.

A schematic showing the fabrication procedure of the PVA/SG hydrogel is illustrated in Scheme 1. The synthesis of the PVA/SG hydrogel was performed as per the previously reported method [25]. Briefly, the PVA hydrogels blended with SG were fabricated by the freezing–thawing method. An aqueous 6 wt% PVA solution was prepared by dissolving PVA into DW in a water bath at 90 °C for 2 h. After, 1 wt% SG solution was prepared and dissolved in deionized water. The PVA solution was mixed at various ratios of SG solution (Table 1). After mixing, each mixture was poured into 24-well plates and underwent repeated freezing–thawing cycles, consisting of 18 h freezing at −20 °C and 6 h thawing at room temperature. The freezing–thawing cycles were prepared under five different conditions to optimize the hydrogel’s properties. Preparation of the hydrogel is illustrated in Scheme 1.

### 2.4. Fourier Transform Infrared Spectrum (FTIR)

The FTIR spectra were taken using an FTIR spectrometer (TENSOR27, Bruker, Ettlingen, Germany). A wavenumber range of 4000–6000 cm^−1^ and 16 scans were used to obtain a resolution of 1 cm^−1^.

### 2.5. Field Emission Scanning Electron Microscopy (FE-SEM)

The cross-sectional structures of the PVA/SG hydrogels were observed using FE-SEM (JSM-7800 F Prime, JEOL Ltd., Tokyo, Japan). The hydrogels were frozen for 4 h and then lyophilized for 24 h. The lyophilized samples were coated with platinum at 20 mA for 60 s in vacuum to provide electrical conductivity. Then, the sample was observed at a magnification of 100 times at an acceleration voltage of 5.0 kV. The porosity of the hydrogels was measured using ImageJ software (NIH Image) and expressed as percentage.

### 2.6. X-ray Diffraction (XRD) Measurements

The crystal structures of the PVA, PVA/SG 50/50 hydrogel, and SG were analyzed using an X-ray analytical instrument (Rigaku SmartLab, Akishima, Japan) equipped with a HyPix-3000 detector. The PVA was analyzed in its powder form, and the SG and PVA/SG 50/50 hydrogel samples were prepared by lyophilization before measurement. X-ray diffraction (XRD) patterns within the range 2θ = 10–80° were examined by using CuKα under the tube voltage of 30 kV and the tube current of 20 mA.

### 2.7. Thermogravimetric Analysis (TGA)

The thermal performance of the PVA, PVA/SG 50/50 hydrogel, and SG was evaluated by thermogravimetric analysis (TGA) using a thermogravimetric analyzer (NETZSCH STA449F3, Selb, Germany) under the temperature of 30–600 °C with the heating rate of 10 °C/min and an N_2_ flow rate of 50 mL/min.

### 2.8. Differential Scanning Calorimetry (DSC)

The thermal behavior of the PVA, PVA/SG 50/50 hydrogel, and SG were assessed using a DSC 7020 (SEICO INST). Before heating under nitrogen, 10 mg of the sample was put in a sealed aluminum pan. The observations were recorded over a temperature range of 0–230 °C at a scanning rate of 10 °C min^−1^.

### 2.9. Rheological Measurements

The rheological properties of the PVA/SG hydrogels were investigated by oscillation angular frequency sweep and oscillatory strain amplitude sweep tests using a DHR-2 rheometer (TA Instruments, New Castle, DE, USA). The angular frequency sweep test was performed from 0.1 to 100 rad/s at a strain of 0.5% at 25 °C. The strain amplitude sweep test was performed from 0.1% to 100% maximum strain at 1.0 Hz. The rheological properties of all hydrogels were measured using a 20 mm parallel plate.

### 2.10. Compressive Tests

Compressive tests were conducted using a Micro-fatigue Tester E3000LT (Instron Inc., Norwood, MA, USA). The compressive tests were performed on hydrogels with a height of 10 mm and a diameter of 15 mm at a rate of 10 mm/min.

### 2.11. Swelling Behavior

To measure the swelling ratio of the hydrogels, each of the dried PVA/SG hydrogels was equilibrated by immersion in deionized water at 25 °C. Then, the swollen hydrogels were weighed several times according to each time. The swelling ratio was calculated by the formula:

$$\mathrm{Swelling}\;\mathrm{ratio}\;(g/g)=\frac{{(W}_{t}-W_{0})}{W_{0}}$$

where *W*_0_ and *W_t_* denote the weight of the dried and swelling hydrogels at regular time intervals, respectively. All experiments were performed in triplicate (*n* = 3) with ±SD.

### 2.12. Drug Release

The 5-FU release patterns of the hydrogels were evaluated in PBS buffers (pH 7.4 and 1.2) at 37 °C. Approximately 0.5% of the volume in the release solution was periodically withdrawn from the PBS. The total solution volume was maintained continuously by adding new PBS. A calibration curve was prepared using the intensity measured according to the 5-FU concentration (0.625, 1.25, 2.5, 5, and 10 mM) with a UV spectrophotometer UV2450 (Shimadzu Corporation, Kyoto, Japan) at 266 nm. The cumulative amount of 5-FU was determined by a UV spectrophotometer at 266 nm using the following equation:

$$\begin{array}{c} \mathrm{Cumulative}\;\mathrm{amount}\;\mathrm{of}\;\mathrm{the}\;5-\mathrm{FU}\\ =C_{n}V+\sum_{i=1}^{i=n-1}C_{i}V_{i} \end{array}$$

where *V* is the release of the solution volume, *V_i_* is the sampling volume, and *C_n_* and *C_i_* are the concentration of 5-FU in the release solution and the extraction sample, respectively. The experiment was performed in triplicate (*n* = 3) with ±SD.

### 2.13. In Vitro Cytotoxicity Test

The cytotoxicity of the PVA/SG hydrogel was evaluated by the WST-8 assay of human embryonic kidney 293 cells (HEK-293 cells, Bank of Korea cell line, Seoul, Republic of Korea) [26]. For the direct cytotoxicity tests, the HEK-293 cells were seeded into 24-well culture plates (Costar, Cambridge, MA, USA) at a concentration of 2 × 10^4^ cells per well with minimum essential medium (MEM, WELGENE, Gyeongsan-si, Republic of Korea) containing 10% fetal bovine serum and 1% penicillin/streptomycin. Then, 5 mg of the hydrogels was added to the wells, and the plates were incubated at 37 °C in a 5% CO_2_ atmosphere. The MEM medium without the PVA/SG hydrogel was used as a negative control, and the MEM medium with 10% (*v*/*v*) dimethylsulfoxide (DMSO) was used as a positive control. After incubation for 2 to 3 days, the WST-8 assay reagent (QuantiMax, BIOMAX, Seoul, Republic of Korea) was added to each well. Absorbance was measured at 450 nm by a SpectraMax ABS Plus Microplate Reader (Molecular Devices, Sunnyvale, CA, USA). Cell viability was determined using the following formula:

$$\mathrm{Cell}\;\mathrm{viability}\;\left(\%\right)=\frac{AbsorbanceofcellswithPShydrogels}{Absorbanceofnegativecontrolcells}$$

The experiment was performed in triplicate (*n* = 3) with ±SD.

## 3. Results

### 3.1. Optimization of the PVA/SG Hydrogel

There were several factors to consider in the development of a hydrogel as a drug delivery system. The swelling rate has been considered one of the most important factors in selection because the degree of drug encapsulation and release can be controlled by the degree of swelling.

#### 3.1.1. Swelling Studies

**Figure 1:**
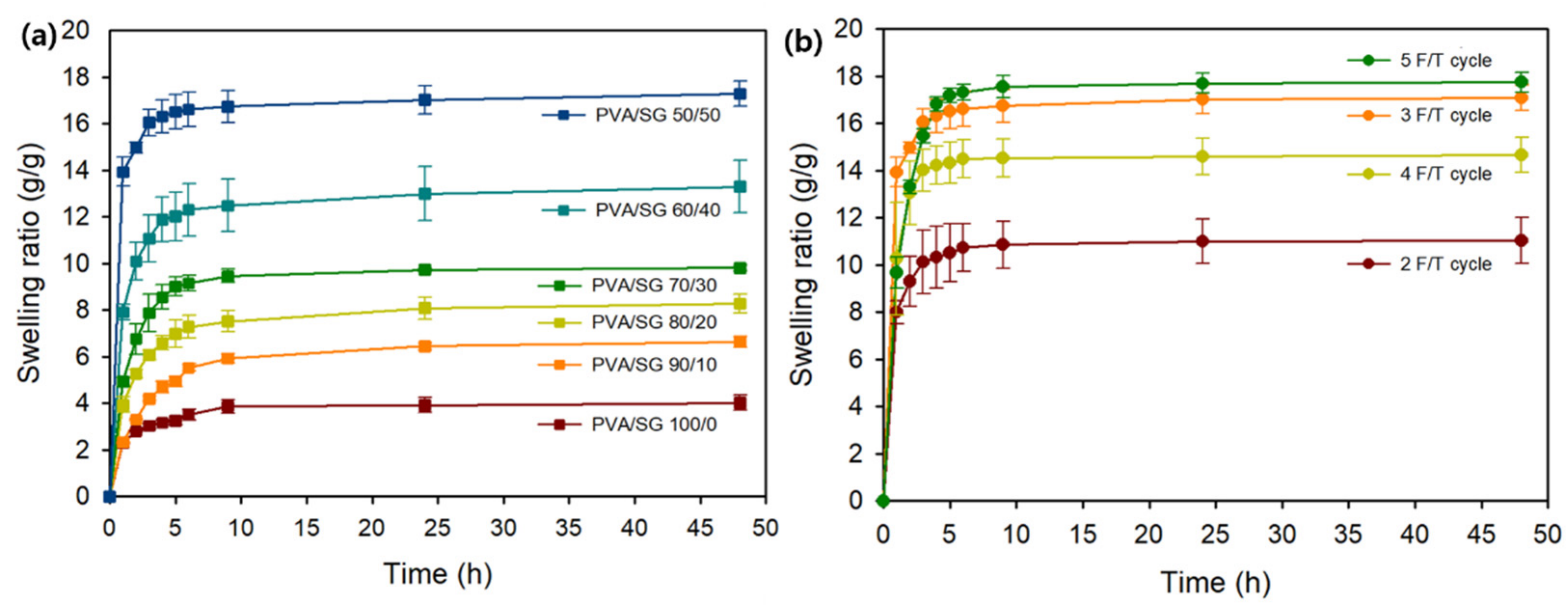
Swelling ratios of the PVA/SG hydrogel under different conditions: (**a**) SG content and (**b**) F/T cycles. The mean values are presented with ±SD (*n* = 3).

The swelling properties of the hydrogels with different SG contents were evaluated under distilled water conditions. The swelling behavior of the hydrogels over time is shown in Figure 1. All of the hydrogels rapidly swelled within 2 h and took about 48 h for the hydrogel to swell fully in distilled water and reach equilibrium. In the equilibrium state, the maximum swelling ratio (MSR) of the PVA/SG 100/0 hydrogel was 4.02 g/g. The swelling capacities of the PVA/SG hydrogel enhanced steadily with the increasing ratios of SG content. As the ratio of SG content reached 50%, the MSR was 17.28 g/g in distilled water, which was about four times higher than the PVA/SG 100/0 hydrogel (Figure 1a). The freeze–thaw cycle (F/T cycle) was considered as an important factor in the process of optimizing the hydrogels using PVA [27]. The swelling ratios of the hydrogel were compared by varying the freeze–thaw cycles based on the previous selected condition.

To compare the swelling rate according to the F/T cycles, five different conditions were prepared. The swelling behavior of the hydrogel was shown with different F/T cycles varying one to five (Figure 1b). The hydrogel with one F/T cycle could not form a rigid hydrogel sufficient enough to measure the swelling ratio (data not shown). The swelling behavior of the hydrogels improved with the increasing number of F/T cycles, followed by a drop three to four F/T cycles. The decrease in the swelling rate at four F/T cycles could be explained by the formation of dense structures that prevent proper bonding of the excess hydrogel network and reduce the swelling rate [28]. According to a previous report, it could be confirmed that this tendency was attributed to phase separation [29]. As the number of F/T cycles increased, physical crosslinking formed through the interaction of the PVA and the SG polymer. This interaction could generate a three-dimensional network structure, improving the swelling rate. It was difficult to form a hydrogel structure at room temperature because each PVA and SG polymer had a short contact time. When the polymer solution was placed at −20 °C, known as the freeze state, the solution was frozen, and adjacent polymer chains created a crystalline structure via hydrogen bonding that primarily stabilized the bonds between the PVA and SG molecules. After that, during the thawing process, the polymer solution melted and water molecules moved freely, but the microcrystalline structures that formed during the freeze state remained intact. Repeated freezing and thawing processes continuously produced more microcrystalline structures and eventually formed the following porous hydrogel network [30]. By repeating the freeze–thaw steps, PVA-rich regions were formed. The SG chain moved to water-rich regions. It created a larger pore size and enhanced the elasticity of the hydrogel [31].

#### 3.1.2. FE-SEM Analysis

**Figure 2:**
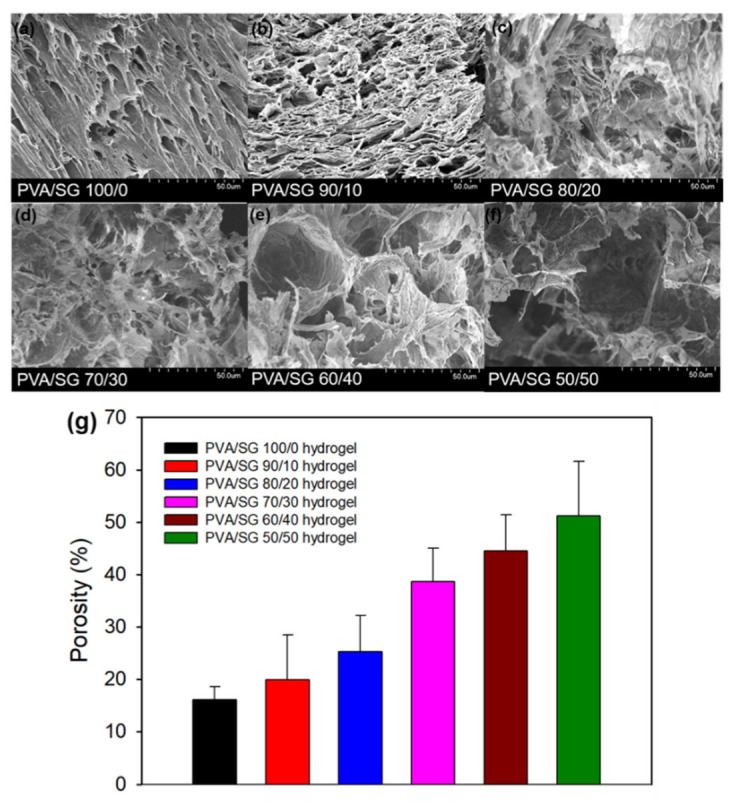
SEM images of the PVA/SG hydrogel according to the ratios of (**a**) 100/0, (**b**) 90/10, (**c**) 80/20, (**d**) 70/30, (**e**) 60/40, and (**f**) 50/50, and (**g**) porosity measurements of the PVA/SG hydrogels. The data are presented as the mean ± SD.

The morphology of the PVA/SG hydrogels were investigated with FE-SEM to evaluate the correlation between pore size and swelling ratio (Figure 2). It was confirmed that the pore size changed depending on the polymer ratios. It was observed that the pore size gradually increased and the uniformity of the pores disappeared with the addition of SG. This was explained by the water-uptake capacity of the SG polymer, which contained a large amount of water on the hydrogel network. As the amount of water in the hydrogel increased, the region occupied by water expanded and pores were constructed by the evaporation of water. These phenomena have been reported in previous studies on the correlation between high-water capacity and the pore size of hydrogels [32,33,34].

#### 3.1.3. Rheological Analysis

**Figure 3:**
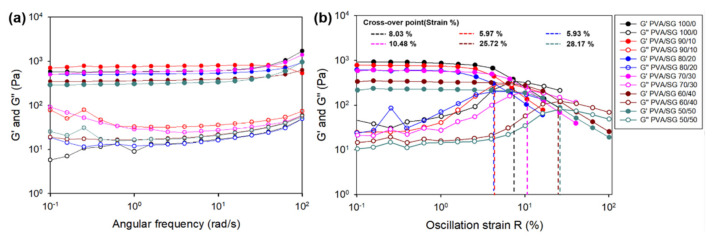
Storage modulus (G′) and loss modulus (G″) versus (**a**) angular frequency (0.1 to 100 rad/s) at 0.5% strain and (**b**) strain amplitude (R = 0.1% to 120%) at a constant angular frequency (1 Hz).

The oscillation frequency sweeps were conducted with the range of 0.1–100 rad/s at a shear strain of 0.5% to measure the viscoelastic properties of the hydrogels. All PVA/SG hydrogels exhibited higher G′ values than G″ values, suggesting that the form of these hydrogels existed in the gel state during the measurement (Figure 3a). In the viscoelastic region from 0.1% to 1%, the G’ value of the hydrogel showed a tendency of increasing with the increase in the PVA concentration (Figure 3b). It was revealed that this tendency correlated to the swelling ratio of the hydrogel because the compact network of the PVA without polysaccharides could be broken by the incorporation of SG. The cross-over point of the hydrogels suggested that the gel network was disrupted and converted from a gel-state to a sol-state at this strain. The PVA/SG 50/50 hydrogel showed the highest cross-over point, indicating that the PVA/SG 50/50 hydrogel had the best elasticity and flexibility among the measured hydrogels. These results were consistent with previous reports that hydrogels with high elasticity and flexibility had high swelling capacities [35,36].

#### 3.1.4. Compressive Test

**Figure 4:**
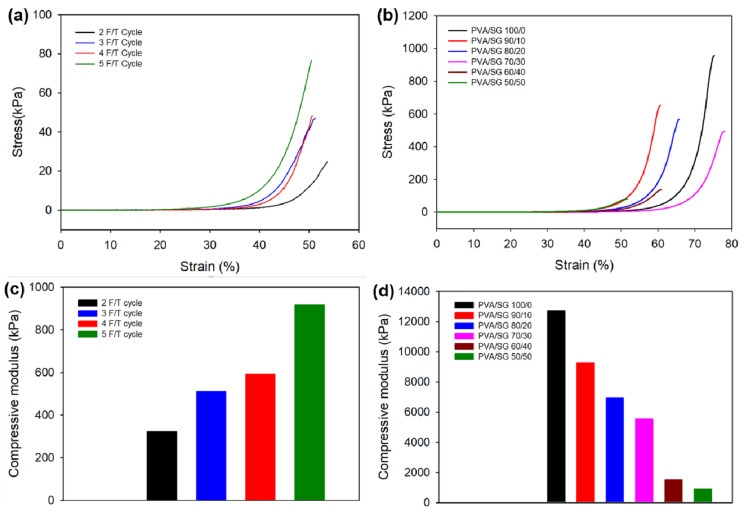
The compressive stress–strain curves of the hydrogel with varying (**a**) F/T cycles and (**b**) polymer concentrations. Compressive value of the hydrogels with varying (**c**) F/T cycles and (**d**) polymer concentrations.

The compressive stress–strain curves and compressive modulus of the hydrogels with varying F/T cycles and polymer concentrations are presented in Figure 4. Primarily focusing on the stress–strain curves, all hydrogel samples had “J”-shaped curves, indicating the high compressive strength [37]. The compression stress of the hydrogel gradually increased with additional freeze–thaw cycles at 50% strain. A similar pattern of correlation between the compressive modulus and freeze–thaw cycles was verified with various PVA polymer concentrations under five freeze–thaw cycles [12]. It was also confirmed that as the ratio of SG increased, the value of the compressive stress decreased. This could be surmised from the correlation between the swelling ratio and the decrease in the compressive stress. The mechanical properties were inversely proportional to the swelling degree because of the loosening of the network of hydrogels [38]. The same tendency was also observed in the compressive modulus of the hydrogel.

### 3.2. Characterization of the PVA/SG Hydrogel

The PVA/SG 50/50 hydrogel with five freeze–thaw cycles was selected based on the maximum swelling ratio. Characterization of the hydrogel was conducted to investigate the conformational change in the hydrogel before and after gelation. The PVA powder, SG, and PVA/SG 50/50 hydrogel were analyzed.

#### 3.2.1. FTIR Analysis

**Figure 5:**
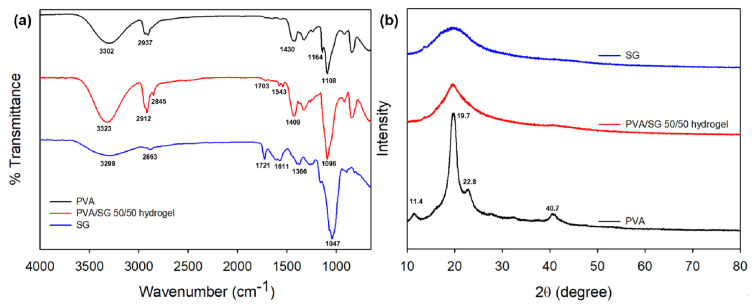
(**a**) FTIR spectra and (**b**) XRD patterns of the PVA, PVA/SG 50/50 hydrogel, and SG.

The FTIR spectra of the hydrogel are shown in Figure 5a. To compare the characteristic peak after gelation, pure PVA and SG powder were analyzed as a control. PVA had its own characteristic peaks at 3302 cm^−1^ (stretching vibration of –OH), 2937 cm^−1^ (asymmetric stretching vibrations—CH_2_), 1430 cm^−1^ (stretching vibration of –C=O), 1164 cm^−1^ (crystalline sequence peak of PVA), and 1108 cm^−1^ (stretching vibration of C-O-C) [39,40,41]. The peaks of the SG were found at 3299 cm^−1^(stretching vibration of –OH), 2863 cm^−1^ (asymmetric stretching vibrations—CH_2_), 1721 cm^−1^ (stretching vibration of C=O acetyl ester), 1611 cm^−1^ (asymmetric stretching vibration of COO^−^ succinate and pyruvate), 1366 cm^−1^ (symmetric stretching vibration of COO^−^ succinate and pyruvate), and 1047 cm^−1^ (stretching vibration of C-O-C) [42]. In the case of the PVA/SG 50/50 hydrogel, the 1164 cm^−1^ peak, which indicated the crystalline sequence peak of the PVA, disappeared after gelation. This indicated that the crystallinity of the PVA, which was used to analyze the structural and conformational change of the PVA, disappeared and the degree of crystallinity of the PVA decreased after gelation [43,44]. New peaks were observed at 1703 cm^−1^ and 1543 cm^−1^, indicating the stretching vibration of the C=O acetyl ester and the symmetric stretching vibration of the COO^−^ succinate and pyruvate with slightly decreased intensities, and the peak shift occurred in the PVA/SG 50/50 hydrogel. It was revealed that the SG was successfully incorporated with the PVA network, while maintaining its own properties.

#### 3.2.2. XRD Studies

Figure 5b shows the XRD patterns of the PVA, PVA/SG 50/50 hydrogel, and SG. As reported in previous papers, PVA is known to have a semi-crystalline structure [45,46]. Its characteristic peaks appeared at 2θ = 11.4°, 19.7°, 22.8°, and 40.7°, representing the typical patterns of crystalline atactic PVA [47]. The peak at 19.5° with a broad pattern indicated the amorphous structure of the SG [48]. After fabricating the hydrogel with SG and PVA, the intensity of the peak at 19.7° decreased because of the interaction of the hydrogen bond between the SG and PVA. This was explained by the increased intermolecular interaction between the SG and PVA, which thereby reduced the crystallinity of PVA network [49].

#### 3.2.3. TGA

**Figure 6:**
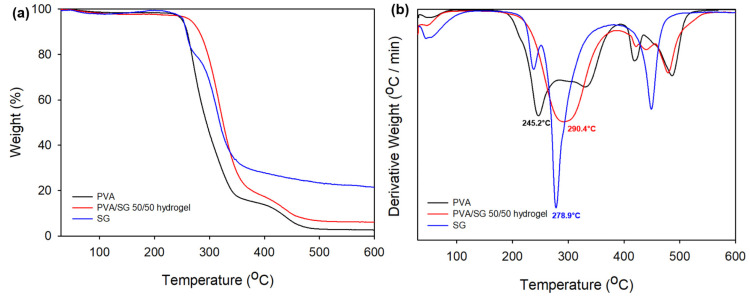
(**a**) TGA and (**b**) DTG curve of the PVA, PVA/SG 50/50 hydrogel, and SG.

To investigate the thermal stability of the hydrogel, the TGA and DTG curves were measured at temperatures from 20 °C to 600 °C (Figure 6). All samples underwent three stages of degradation: (1) evaporation of the water step, (2) dehydration, decomposition, and chain breakage of the polymers, and (3) degradation of the polymer by-product. Degradation mainly occurred in the second step. From the DTG curve, the maximum temperatures of the PVA measured under 250 °C, while those for the other sample, which was composed with SG, were over 270 °C. It has been suggested that the thermal stability of SG-containing samples is enhanced by inducing an effective delay during thermal decomposition [50]. It was also confirmed that the final remaining weight percentages of the samples were calculated to be about 21% (SG), 6% (PVA/SG 50/50 hydrogel), and 2% (PVA). These results corresponded with previous reports that the thermal stability of hydrogels was enhanced after incorporating SG [51].

#### 3.2.4. DSC

**Figure 7:**
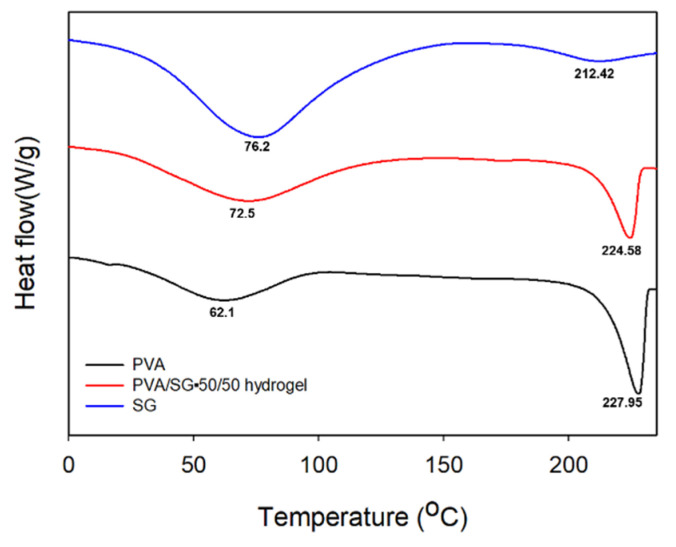
DSC curves of the PVA, PVA/SG 50/50 hydrogel, and SG.

To further investigate thermal stability, a DSC analysis of samples was performed. As shown in Figure 7, the glass transition temperature (T_g_) of the PVA is known to be about ~86 °C, but 62.1 °C was measured during this experiment. This decrease in T_g_ was due to the effect of moisture in the sample [52,53]. The melting temperature (T_m_) of the PVA was 227.95, which was due to the melting of the PVA crystalline correlated with previous reports [54,55,56]. The T_g_ of the SG was measured at 76.2 °C, and the T_m_ was 212.42. After incorporating PVA and SG, the T_g_ values of the PVA/SG 50/50 hydrogel shifted near to the median value of the PVA and SG, indicating that PVA and SG were well blended to form a homogenous PVA/SG hydrogel network and that they enhanced the thermal stability compared with PVA [57]. The T_m_ value of the PVA/SG 50/50 hydrogel slightly shifted compared to that of the PVA, which provided evidence for the effect on the crystallization of the PVA [58].

### 3.3. In Vitro Drug Release and Cell Viability

#### 3.3.1. pH-Dependent Drug Release of 5-FU

**Figure 8:**
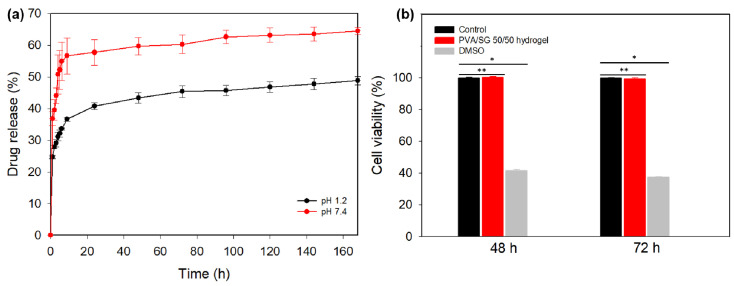
(**a**) 5-FU release profile with different condition at pH 1.2, 7.4 (**b**) Cell viability (%) of HEK-293 cell cultured with hydrogels for 48, 72 h. DMSO was a positive control. Mean values presented with ±SD (*n* = 3). * indicates significant difference (*p* < 0.05), ** (*p* < 0.005).

The release pattern was shown in Figure 8a. Under both conditions, it was shown that the drug encapsulated in the hydrogel released rapidly within 12 h. This rapid release pattern could be explained by the fact that most of the 5-FU was present on the surface of the hydrogel, resulting in rapid release due to diffusion by the concentration difference between the hydrogel and the buffer solution in the initial release state [59]. After 3 days soaked under the two conditions, the amount of the drug released did not increase remarkably, and it showed a steady pattern. The final drug release rates were 64.5% at acidic condition and 48.8% at slightly basic condition. These results could be explained by the protonation of the carboxylated ion in the SG. The functional groups on the SG were composed of succinate, pyruvate, and acetate. The pK_a_ values of each group were 2.45 for pyruvate, 4.21 succinate, and 4.76 acetate. At pH 1.2, which was below pK_a_, all of the functional groups remained in the –COOH form. This induced intermolecular hydrogen bonding between the SG molecules, so it interrupted the movement of the water molecules. The encapsulated 5-FU in the hydrogel could not diffuse well because of strong hydrogen bonding. At pH 7.4 (above pKa), electrostatic repulsion between the functional groups on the SG occurred. The intermolecular force of the network was weakened because of repulsion; thus, it could provide a comfortable condition to move out of the buffer conditions [60,61,62].

#### 3.3.2. Cell Viability

It is well-recognized that materials with good biocompatibility are an essential consideration for use in the biomedical field [63]. The human embryonic (HEK-293) cell was investigated as a model cell line for the cytotoxicity test for the hydrogels. As shown in Figure 8b, the PVA/SG 50/50 hydrogel showed no cytotoxicity effect on the HEK-293 cell. According to GB/T 16886.5-2003 (ISO 10993-5: 1999), samples with cell viability greater than 75% can be considered as non-cytotoxic. PVA has been widely used in the biomedical field because of its high biocompatibility, with the acute oral toxicity of PVA also highlighted (LD_50_ = 15–20 g/kg) [64]. SG has also been confirmed as a biocompatible polymer in previously reported papers [65,66].

## 4. Conclusions

A novel hydrogel was developed through the freeze–thaw method by physically blending PVA with SG isolated directly from *Sinorhizobium meliloti* Rm 1021. The PVA/SG hydrogels were optimized to maximize their swelling rate for effective drug encapsulation by adjusting the ratio of PVA and SG in the hydrogels and the freeze–thaw cycles. The maximum swelling rate of 17.28 g/g was obtained when the composition of the PVA and SG was 50:50 (PVA/SG 50/50) and the total number of freeze–thaw cycles was five. The swelling pattern of the hydrogel was explained by SEM with the largest pores and rheological analysis with the highest cross-over point (28.17%) between the storage modulus (G′) and the loss modulus (G″) among the hydrogels. Changes in the conformation and structure of the PVA/SG 50/50 hydrogel were proven by FTIR, XRD, DSC, and TGA. The crystalline structure of the PVA was destroyed due to SG’s introduction, as examined by FTIR and XRD analysis. Furthermore, the PVA/SG 50/50 hydrogel showed improved thermal stability, owing to its interaction with thermally stable SG chains. The improvement in thermal stability was confirmed by the increase in the remaining weight from 2% to 6% after incorporation with the SG through the TGA results. DSC also revealed that the thermal stability of the hydrogel was enhanced based on the results with the shift in the glass transition temperature (T_g_) and melting temperature (T_m_). Furthermore, the PVA/SG 50/50 hydrogel showed differential drug release according to the corresponding pH under acidic conditions of pH 1.2 and slightly basic conditions of pH 7.4. No cytotoxicity was found in the PVA/SG 50/50 hydrogel either, suggesting that the hydrogel could be a potential biomaterial capable of pH-responsive drug delivery for chronic wound dressing applications.

## Figures and Tables

**Table 1:** Composition of the PVA/SG hydrogels.

PVA/SG Hydrogel	6 wt% PVA (mL)	1 wt% SG (mL)
PVA/SG 100/0	1	0
PVA/SG 90/10	0.9	0.1
PVA/SG 80/20	0.8	0.2
PVA/SG 70/30	0.7	0.3
PVA/SG 60/40	0.6	0.4
PVA/SG 50/50	0.5	0.5

## Data Availability

Not applicable.
